# Vitamin D is Positively Associated with Bone Mineral Density Muscle Mass and Negatively with Insulin Resistance in Senile Diabetes Mellitus

**DOI:** 10.1155/2022/9231408

**Published:** 2022-03-29

**Authors:** Yan Gao, Zhaoyu Chen, Zijian Ma

**Affiliations:** ^1^Department of Senior Official Ward, China-Japan Friendship Hospital, Beijing, China; ^2^Arthritis Clinic and Research Center, Peking University People's Hospital, Beijing, China; ^3^Department of Senior Official Ward, The Third Hospital of Hebei Medical University, Shijiazhuang, China

## Abstract

**Objective:**

To explore the correlations between vitamin D level and bone mineral density (BMD), insulin resistance (HOMA-IR), and muscle mass in patients with senile type 2 diabetes mellitus (T2DM).

**Methods:**

Totally, 80 patients with senile T2DM admitted to China-Japan Friendship Hospital from Jan 2020 to Oct 2021 were enrolled and assigned to the 25 (OH)D-deficiency group (*n* = 35) or 25(OH)D-normal group (*n* = 45) according to serum 25(OH)D level. BMD and HOMA-IR in the femur neck and muscle masses of upper and lower limbs were compared between the two groups, and Pearson's correlation analysis was performed to determine the relations between 25(OH)D and BMD, HOMA-IR, and muscle masses of upper and lower limbs.

**Results:**

No notable difference was found between the two groups in general data including age, gender, diabetes duration, BMI, HBA 1c, and fasting insulin (all *P* > 0.05). Compared with the 25(OH)D-normal group, the 25 (OH)D-deficiency group showed a notably lower BMD in the femur neck, notably lower muscle masses of upper and lower limbs, and a notably higher HOMA-IR level (all *P* < 0.05). The Pearson's correlation analysis revealed positive associations between 25(OH)D and BMD and muscle masses of upper and lower limbs in patients with senile T2DM and a negative correlation between 25(OH)D and HOMA-IR (all *P* < 0.05).

**Conclusions:**

The serum 25(OH)D decreases notably in patients with senile T2DM, and higher serum 25(OH)D level may improve insulin resistance, limb muscle masses, and bone density and thus maintain bone health.

## 1. Introduction

With the rapid development of social economy, the change of diet structure, and the emergence of aging trend, senile type 2 diabetes mellitus (T2DM) shows an annually growing incidence, seriously compromising patients' health and quality of life [1]. Among Chinese patients with senile T2DM, the incidence of sarcopenia reaches 21%, which hinders the physical activity and daily living ability, undermines the quality of life, and increases the hospitalization rate, medical expenses, and the risk of fractures and chronic cardiopulmonary diseases among the elderly population [2]. Patients with senile T2DM are prone to osteopenia or osteoporosis, with a prevalence of osteoporosis of 31.7% [3]. The incidence of T2DM is associated with insulin resistance (IR) induced by decreased insulin sensitivity and insufficient insulin secretion induced by islet cell destruction and dysfunction [4]. Vitamin D is a fat-soluble vitamin and plays a key role in the development of bone, maintenance of bone mineralization and regulation of the metabolism of calcium and phosphorus. Being strongly correlated with the development and progression of T2DM, vitamin D can increase insulin sensitivity, improve blood sugar control, and contribute to prevent and reduce some diabetic complications [5]. To our knowledge, vitamin D can increase the body's absorption of calcium and phosphorus and balance the calcium and phosphorus, and its deficiency can cause bone metabolic disorder, thus resulting in a decline in bone mass and bone loss. Additionally, vitamin D also plays a role in relieving stress, restraining activity of RAAS system, regulating cell apoptosis, maintaining immunity, reducing urinary protein, and improving insulin resistance. It is reported that 25(OH)D serves as an optimal indicator to evaluate the status of vitamin D and is closely related to the occurrence and disease control of T2DM via promoting the synthesis and secretion of insulin [6]. Nevertheless, more evidence-based medical evidence is urgently required to clarify the specific relation between serum vitamin D and bone mineral density, muscle mass, and insulin resistance in senile diabetes. To fill this gap, this study evaluated the changes of serum vitamin D in patients with senile T2DM and analyzed the correlation between serum vitamin D and IR, bone mineral density BMD, and muscle mass.

## 2. Data and Methods

### 2.1. General Data

Totally, 80 patients with senile T2DM admitted to our hospital from Jan 2020 to Oct 2021 were enrolled. The written consent was obtained from all participants before enrolment. The protocol of this study was approved by the ethic committee of the China-Japan Friendship Hospital with the approved No. of 2019-11/23.

### 2.2. Inclusion and Exclusion Criteria

#### 2.2.1. Inclusion Criteria

The inclusion criteria are as follows: (1) aged ≥60 years old; (2) met the diagnostic criteria of the World Health Organization (WHO) for the diagnosis of T2DM [7]; and (3) participated in the study voluntarily.

#### 2.2.2. Exclusion Criteria

The exclusion criteria are as follows: (1) with comorbid diabetic ketoacidosis or diabetic ketosis; (2) with severe dysfunction of important organs such as heart, liver and kidney, acute infection, or tumour; and (3) had taken vitamin D for a long time.

### 2.3. Methods

#### 2.3.1. General Data of Patients

All subjects were inquired by the same doctor for medical history and given physical examination by the same doctor. The body mass index (BMI) of patients was calculated, and their age, weight, height, gender, and course of disease were all recorded.

#### 2.3.2. BMD

The BMD in the left femoral neck of each subject was measured by an American OSTEOMETER dual-energy X-ray absorptiometer (DTX-2000).

#### 2.3.3. Muscle Mass

The muscle masses of upper and lower limbs were determined using a dual-energy X-ray absorptiometer.

#### 2.3.4. Specimen Collection and Determination

Fasting venous blood was collected from each subject, followed by quantification of serum 25(OH)D via the enzyme-linked immunosorbent assay (ELISA). Based on the serum 25 (OH)D level, the patients were assigned to the 25 (OH)D-deficiency group (25 (OH)D < 70 nmol/L) or the 25 (OH)D-normal group (25(OH)D ≥ 70 nmol/L). The fasting insulin (FINS, *μ*U/mL) was measured by the chemiluminescence immunoassay, and fasting plasma glucose (FPG, mmol/L) by the glucose oxidase method. HOMA-IR [8] was calculated according to the following formula: HOMA − IR = FINS × FPG/22.5, where 22.5 is the correction factor. The calculation formula indicates that 5 *μ*U/mL plasma insulin corresponds to 4.5 mmol/L blood glucose level in a normal ideal individual. All determinations were conducted in accordance with the laboratory quality control standards.

### 2.4. Statistical Analysis

All data analysis was performed with SPSS 24.0. The continuous variables were expressed as mean with standard deviation (*x̅*±*s*) and Shapiro-Wilk test was adopted to check the normal or nonnormal distribution. For normal distribution continuous variables, intergroup comparison was conducted via the independent-samples *t* test, while the intragroup comparison was conducted via the pair-sample *t* test. For nonnormal distribution continuous variables, *t* test was adopted after normal transformation. The categorical data was expressed as cases with percentage (*n*, %), and the difference was tested and compared using a chi-squared test. Pearson's correlation analysis was conducted for analysing the associations between 25(OH)D and BMD and HOMA-IR and muscle masses of upper and lower limbs. The conventional *P* ≤ 0.05 was used to assess statistical significance.

The difference was tested and compared using a chi-squared test,

## 3. Results

### 3.1. General Data of 25(OH)D-Deficiency Group and 25(OH)D-Normal Group

In the 25(OH)D-deficiency group, the mean age was (71.42 ± 8.74) years, the course of disease was (6.54 ± 3.61) years, the BMI was (23.42 ± 3.21), the FPG was (8.56 ± 2.47) mmol/L, and the ratio of male to female was 16: 19; in the 25(OH)D-normal group, the mean age was (69.58 ± 11.03) years, the course of disease was (5.62 ± 3.33) years, the BMI was (23.52 ± 4.40), the FPG was (7.97 ± 2.31) mmol/L, and the ratio of male to female was 24: 21. No notable difference was found between the two groups in general data, fasting insulin level, HbA1c, diabetes duration, and the frequency of coexisting diseases (*P* < 0.05, Table 1).

### 3.2. The BMD, Muscle Mass, and HOMA-IR in 25(OH)D-Deficiency Group and 25(OH)D-Normal group

The femoral neck BMD of the two groups was shown in Table 2. The femoral neck BMD in the 25(OH)D-deficiency group was (0.72 ± 0.13) g/cm^2^, while (0.81 ± 0.06) g/cm^2^ in the 25(OH)D-normal group. The 25(OH)D-deficiency obtained a notably lower femoral neck BMD than the 25(OH)D-normal group (*P* < 0.05, Table 2). Femoral neck BMD is a strong predictor of hip fracture, suggesting that the 25(OH)D-deficiency group is prone to hip fracture.

In the 25(OH)D-deficiency group, the upper limb muscle mass was (4.39 ± 0.22) kg and the lower limb muscle mass was (14.78 ± 0.73) kg. In the 25(OH)D-normal group, the upper limb muscle mass was (4.77 ± 0.31) kg and the lower limb muscle mass was (15.67 ± 0.68) kg. The 25(OH)D-deficiency group showed notably lower muscle masses of upper and lower limbs than the 25(OH)D-normal group (*P* < 0.05, Table 2). Muscle mass is important for muscle strength and quality of life, and lower muscle mass indicates a lower muscle strength in the 25(OH)D-deficiency group.

The HOMA-IR of the 25(OH)D-deficiency group was 4.85 ± 0.49 and 4.33 ± 0.52 in the 25(OH)D-normal group. The 25(OH)D-deficiency group showed a notably higher HOMA-IR than the 25(OH)D-normal group (*P* < 0.05, Table 2). Higher HOMA-IR indicates elevated insulin resistance levels of the 25(OH)D-deficiency group.

### 3.3. Correlations between Serum 25(OH)D Level and BMD and HOMA-IR and Muscle Mass

The Pearson's correlation analysis revealed positive associations between 25(OH)D and BMD, upper limb muscle mass, and lower limb muscle mass in patients with senile T2DM and a negative relation between 25(OH)D and HOMA-IR (all *P* < 0.05, Table 3).

## 4. Discussion

The vitamin D deficiency witnesses a high prevalence in elderly male population in China, among which the proportions of severe deficiency, and the deficiency and insufficiency are 20.97%, 39.20%, and 24.36%, respectively [9]. Over the past few years, many studies have confirmed the association between T2DM and vitamin D [10]. Hongxia et al. [11] revealed a lower active form of vitamin D level in T2DM patients than health enrolments. Sarcopenia is a muscle-wasting syndrome characterized by progressive and systemic degenerative loss of skeletal muscle mass (SMM) and strength, often associated with aging and many chronic diseases. In addition to muscular dystrophy, people with sarcopenia demonstrate a higher risk of fractures and metabolic diseases, compromising their quality of life. Neurological complications of diabetes and sarcopenia (metabolic disorders of diabetes, hormonal abnormalities, oxidative stress, and inflammatory response) would aggravate muscle damage [12]. Additionally, loss of muscle mass and function plays a negative role in controlling blood sugar and further leads to metabolic disorders.

In the present study, in contrast to the 25(OH)D-normal group, the 25(OH)D-deficiency group showed notably lower BMD in the femur neck, lower muscle masses of upper and lower limbs, and higher HOMA-IR level. Moreover, the Pearson's correlation analysis revealed positive associations between 25(OH)D and BMD and muscle masses of upper and lower limbs in patients with senile T2DM and a negative relation between 25(OH)D and HOMA-IR. Reportedly, the occurrence of T2DM is attributed to IR and insulin secretion dysfunction. Vitamin D level can regulate the synthesis and secretion of insulin and is related to fasting blood glucose, glycosylated hemoglobin, IR, and islet *β* cells. To our best understanding, active form of vitamin D level deficiency lowers the sensitivity of insulin and thus increases IR [13]. Its supplementation can reduce HOMA-IR-associated metabolic parameters and improve glucose and lipid metabolism, and the serum vitamin D level can lower HOMA-IR and reduce related diseases by lowering oxidative stress and improving metabolic status [14]. The mechanism of vitamin D on IR can be explained as follows: (1) vitamin D level affects insulin secretion of pancreatic *β* cells by regulating Ca^2+^ homeostasis and thus improves insulin sensitivity and insulin signal transduction [15]; (2) it controls the Ca^2+^ level in muscle cells and adipocytes to maintain the normal function of insulin responsive tissue, including muscle and adipose [16]; (3) it reduces low-degree chronic inflammation, with indirect antioxidant properties and immunomodulatory function [16]; (4) it controls epigenetic gene expression [17]; and (5) it affects lipid and glucose metabolism in muscle tissue and the liver [18].

The prevalence of fractures in patients with senile T2DM in China is 7.3%, and the decrease in BMD is one of the independent risk factors of fractures [19]. Serum vitamin D level plays a crucial part in bone metabolism by promoting the absorption of calcium and phosphorus to maintain stable blood calcium and phosphorus concentrations and promoting bone mineralization, stimulating osteoblasts to promote bone formation and inhibit osteoblast apoptosis, and promoting the differentiation of precursor osteoclasts into mature osteoclasts and promoting bone resorption. Serum vitamin D deficiency is frequently seen among postmenopausal women, and it is positively associated with BMD in the femoral neck, and appropriate vitamin D supplementation is of profound significance to prevent bone loss [20]. Senile T2DM is often complicated with osteopenia or even osteoporosis, and serum vitamin D deficiency in the body would result in the decrease in BMD [21].

The vitamin D can increase the synthesis of protein in muscle and the absorption of calcium in sarcoplasmic reticulum and influence the growth of muscle system by regulating various factors secreted by the muscle system itself and surrounding tissues [22]. *In vitro* assays revealed positive influences of vitamin D-based therapy on muscle cell hypertrophy, muscle fibers, and muscle strength in cultured cells. A prior study pointed out that vitamin D supplementation can regulate lipid and mitochondrial muscle metabolism and can also directly impact glucose metabolism and insulin-driven signal transduction [23]. For patients with senile T2DM, serum vitamin D level deficiency contributes to the decrease of muscle mass and function and would result in the decrease of lower limb muscle mass [24]. For patients with senile T2DM, a notable decrease of serum 25(OH)D level may trigger IR and decreased BMD and muscle mass. Serum vitamin D and BMD and muscle masses of upper and lower limbs are positively related, while serum vitamin D and HOMA-IR are negatively related. This study confirmed the correlation between vitamin D deficiency and bone mineral density, muscle mass, and insulin resistance, providing evidence-based medical basis for vitamin D supplementation in senile T2DM, but there are still the following problems. First, this study was a single-center study, with a small number of patients included and a lack of representativeness. Secondly, the effects of vitamin D supplementation on bone mineral density, muscle mass, and insulin resistance in patients with vitamin D deficiency were not observed in this study. In the future, we will enlarge the sample size and follow-up time and further explore the effect of vitamin D supplementation.

## 5. Conclusion

The serum 25(OH)D level correlates with bone and muscle mass as well as insulin resistance in senile T2DM patients. Vitamin D deficiency in senile T2DM may accelerate insulin resistance, loss of limb muscle masses, and bone density. Therefore, vitamin D supplementation in senile T2DM may decrease insulin resistance and the risk of bone fracture and loss of muscle mass.

## Figures and Tables

**Table 1:** General data analysis.

	25(OH)D-deficiency group (*n* = 35)	25(OH)D-normal group (*n* = 45)	*t*/*χ*2	*P*
Mean age	71.42 ± 8.74	69.58 ± 11.03	0.809	0.421
Gender			0.457	0.676
Male	16	24		
Female	19	21		
Diabetes duration (years)	6.54 ± 3.61	5.62 ± 3.33	1.182	0.241
BMI (kg/m^2^)	23.42 ± 3.21	23.52 ± 4.40	0.113	0.910
HbA1c (%)	5.58 ± 1.15	5.29 ± 1.84	0.816	0.417
Fasting insulin (mmol/L)	8.56 ± 2.47	7.97 ± 2.31	1.099	8.560
Coexisting diseases				
Hypertension	12	17	0.104	0.322
Coronary artery disease	5	11	1.270	0.260
Hyperlipidaemia	6	10	0.318	0.573

**Table 2:** Comparison of insulin resistance (*x̅*±*s*).

	25(OH)D-deficiency group (*n* = 35)	25(OH)D-normal group (*n* = 45)	*t*	*P*
Femoral neck BMD	0.72 ± 0.13	0.81 ± 0.06	4.112	≤0.001
Upper limb	4.39 ± 0.22	4.77 ± 0.31	5.898	<0.001
Lower limb	14.78 ± 0.73	15.67 ± 0.68	5.617	≤0.001
HOMA-IR	4.85 ± 0.49	4.33 ± 0.52	4.565	≤0.001

**Table 3:** Correlations between 25(OH)D and BMD and HOMA-IR and muscle mass.

	BMD	HOMA-IR	Upper limb muscle mass	Lower limb muscle mass
*r*	0.372	-0.472	0.557	0.520
*P*	≤0.001	≤0.001	≤0.001	≤0.001

## Data Availability

The datasets used during the present study are available from the corresponding author upon reasonable request.
